# Supplementary material for: PiggyBac transposon tools for recessive screening identify B-cell lymphoma drivers in mice
Source: Nat Commun. 2019 Mar 29;10:1415. doi: 10.1038/s41467-019-09180-3 (PMC6440946; doi:10.1038/s41467-019-09180-3)
Supplement: Supplementary file 1 — Supplementary Information [file 41467_2019_9180_MOESM1_ESM.pdf]

Weber, de la Rosa, Grove *et al.* **"*PiggyBac* transposon tools for recessive screening identify B-cell lymphoma drivers in mice"**

Supplementary Information

Supplementary Figures 1 – 19

Supplementary Tables 1 – 4

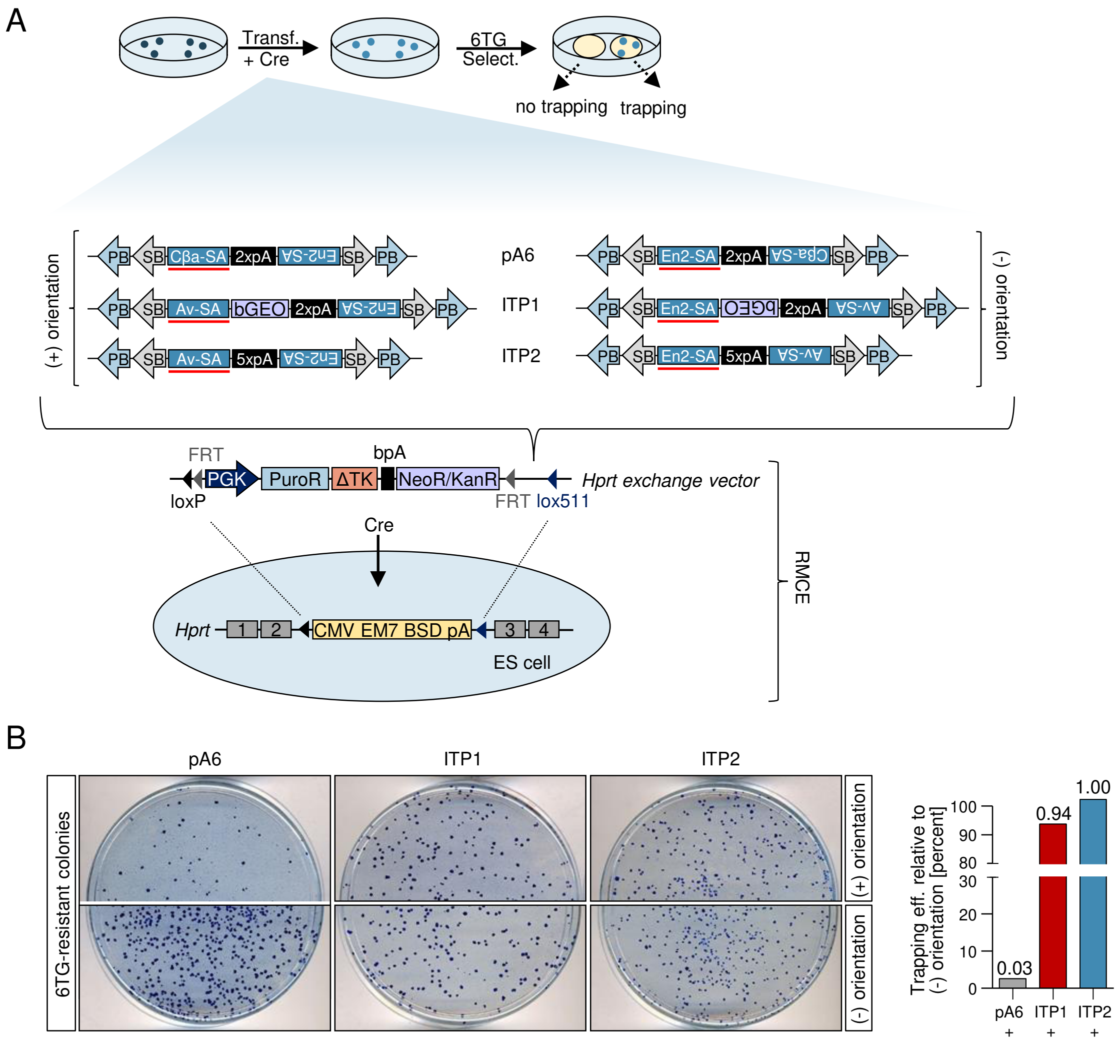

Supplementary Figure 1. Comparison of gene trapping efficiencies mediated by different splice acceptors using RMCE at the *Hprt* locus. (**A**) Transposons carrying different splice acceptors (SA: carp *β-actin* SA [Cβa-SA], adenovirus-derived SA [Av-SA] and mouse *Engrailed-2* exon-2 SA [En2-SA]) were cloned into a *hypoxanthine phosphoribosyltransferase (Hprt)* exchange vector between a loxP and a lox511 cassette in both orientations (plus (+) and minus (-) orientation). These *Hprt* exchange vectors were electorporated individually together with a Cre expression vector into male embryonic stem (ES) cells, which harbor an “acceptor cassette” knock-in at the X-chromosomal *Hprt* locus. Recombinase mediated cassette exchange (RMCE) led to replacement of a resistance marker cassette (CMV-EM7-BSD-pA) with the transposon-containing exchange cassette (elements between the loxP and lox511 cassette in the *Hprt* vector). ES cells were then selected with 6-thioguanine (6TG). Efficient splicing by the SA within the transposon leads to a premature termination of transcription resulting in a non-functional *Hprt* protein. *Hprt* deficiency induces 6TG resistance. (**B**) After selection, cells were stained with crystal violet and 6TG-resistant colonies were counted. The Av-SA and the En2-SA showed comparable gene-trapping efficiencies while Cβa-SA was less efficient in this very sensitive assay (6TG resistance is not compatible with residual “leakiness”). Transf., transfection; Select., selection; PGK, phosphoglycerate-kinase promoter; PuroR, puromycin resistance; ΔTK, truncated herpes simplex virus thymidine kinase; bpA, bovine growth hormone polyadenylation signal; NeoR/KanR, neomycin/kanamycin resistance; FRT, FLP recognition target site; LoxP/Lox511, Cre recognition target site; CMV, cytomegalovirus promoter; EM7, bacterial promoter; BSD, blasticidin resistance; PB, *PiggyBac*; SB, *Sleeping Beauty*; pA, SV40 bidirectional polyadenylation signal; bGEO, β-galactosidase/neomycin resistance reporter; Eff, efficiency.

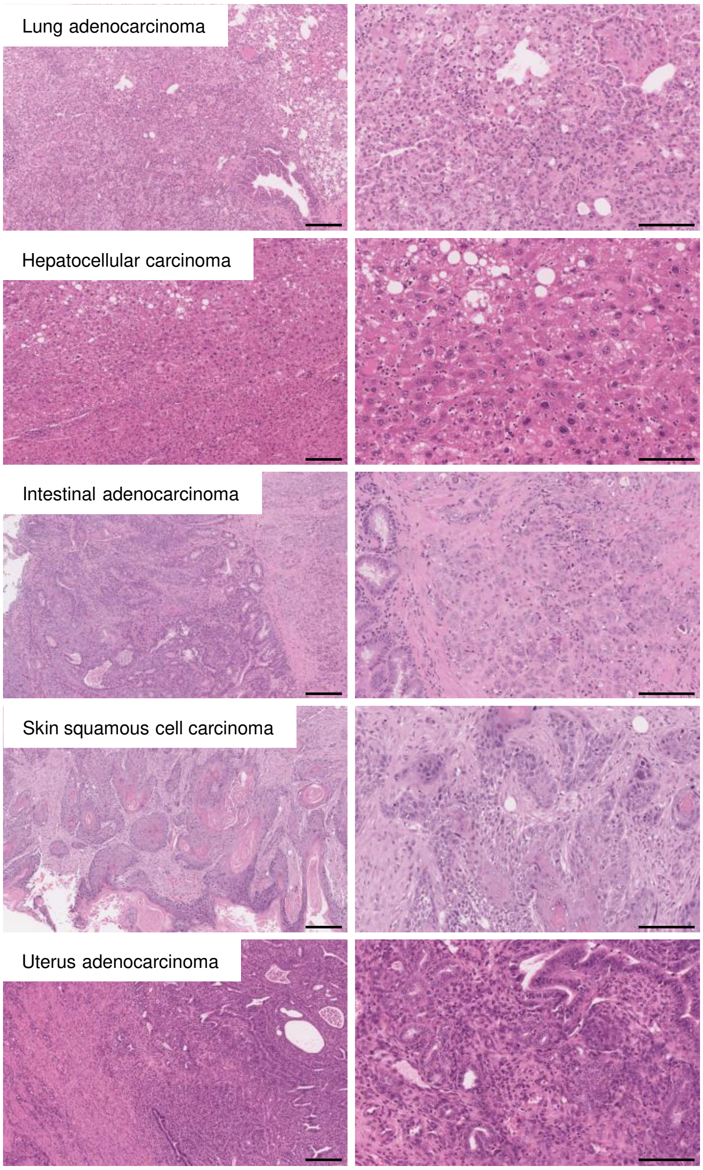

Supplementary Figure 2. Solid tumor development in *IPB* mice. Microscopic images show hematoxylin and eosin stainings of representative solid tumor cases from *ITP2-M;Rosa26*$^{PB/+}$*;Blm*$^{m3/m3}$ *(IPB)* mice. Scale bars: 200 µm (left panel),100 µm (right panel).

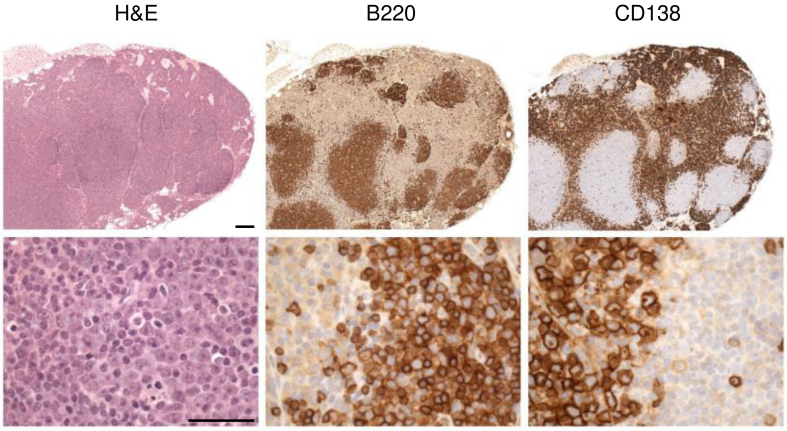

Supplementary Figure 3. DLBCLs in *IPB* mice occasionally show plasmacytic differentiation. Microscopic images show a case of diffuse large B-cell lymphoma (DLBCL) with plasmacytic differentiation. These tumors consist of large-sized neoplastic cells with abundant cytoplasm and a round nucleus with vesicular chromatin and two or more nucleoli (hematoxylin and eosin staining; H&E). A sub-set of tumor cells shows strong expression of plasma cell markers (CD138) and loss of B-cell markers (B220/CD45R). In the representative images above, note the mirror-effect shown by the B220 and CD138 immunohistochemistry, pointing to a transition from mature cells (B220 positive, center of the follicle) to plasmacytoid cells (CD138 positive, follicular periphery). Scale bars: 200 µm (top panel), 50 µm (bottom panel).

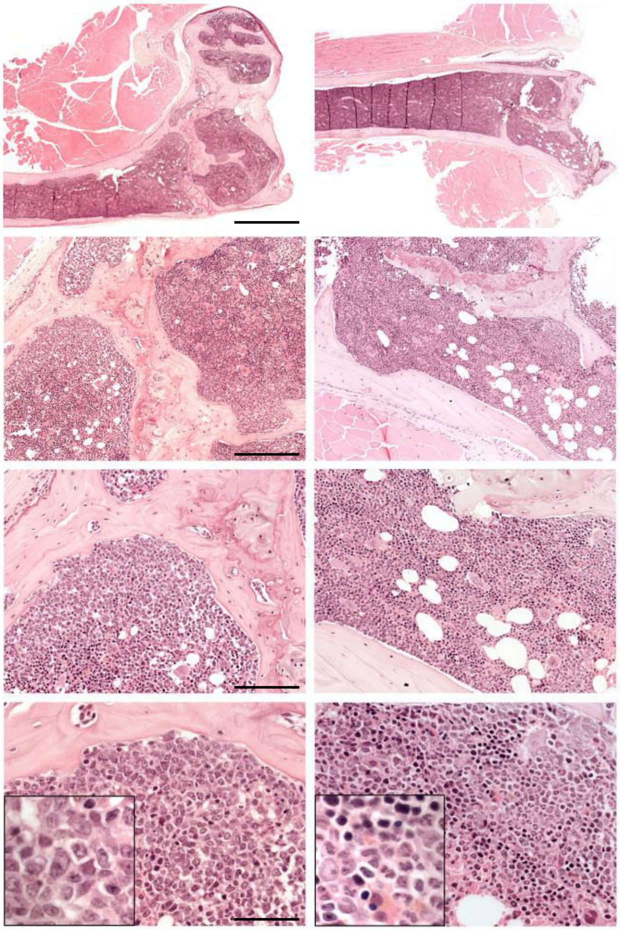

Supplementary Figure 4. **Bone marrow infiltration in *IPB* mice with DLBCL.** Microscopic images (showing hematoxylin and eosin stainings) present diffuse large B-cell lymphoma cases with initial paratrabecular infiltration (left panel) and without (right panel). Scale bars: 1 mm (first row), 200 µm (second row), 100 µm (third row) and 50 µm (last row). Inserts in the last row are in 630x magnification.

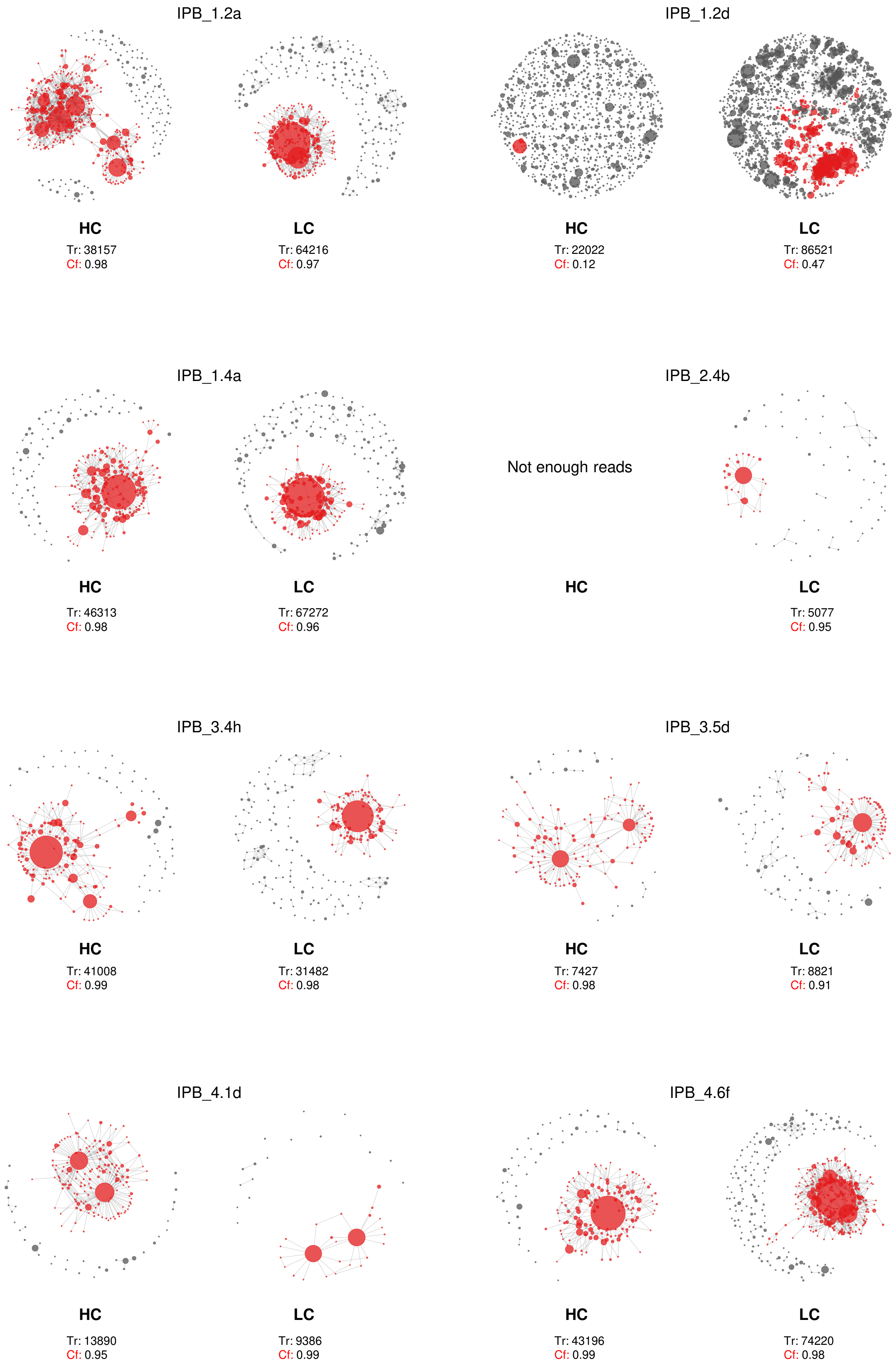
IPB_1.2a
HC
Tr: 38157
Cf: 0.98
LC
Tr: 64216
Cf: 0.97
IPB_1.2d
HC
Tr: 22022
Cf: 0.12
LC
Tr: 86521
Cf: 0.47
IPB_1.4a
HC
Tr: 46313
Cf: 0.98
LC
Tr: 67272
Cf: 0.96
IPB_2.4b
Not enough reads
HC
LC
Tr: 5077
Cf: 0.95
IPB_3.4h
HC
Tr: 41008
Cf: 0.99
LC
Tr: 31482
Cf: 0.98
IPB_3.5d
HC
Tr: 7427
Cf: 0.98
LC
Tr: 8821
Cf: 0.91
IPB_4.1d
HC
Tr: 13890
Cf: 0.95
LC
Tr: 9386
Cf: 0.99
IPB_4.6f
HC
Tr: 43196
Cf: 0.99
LC
Tr: 74220
Cf: 0.98

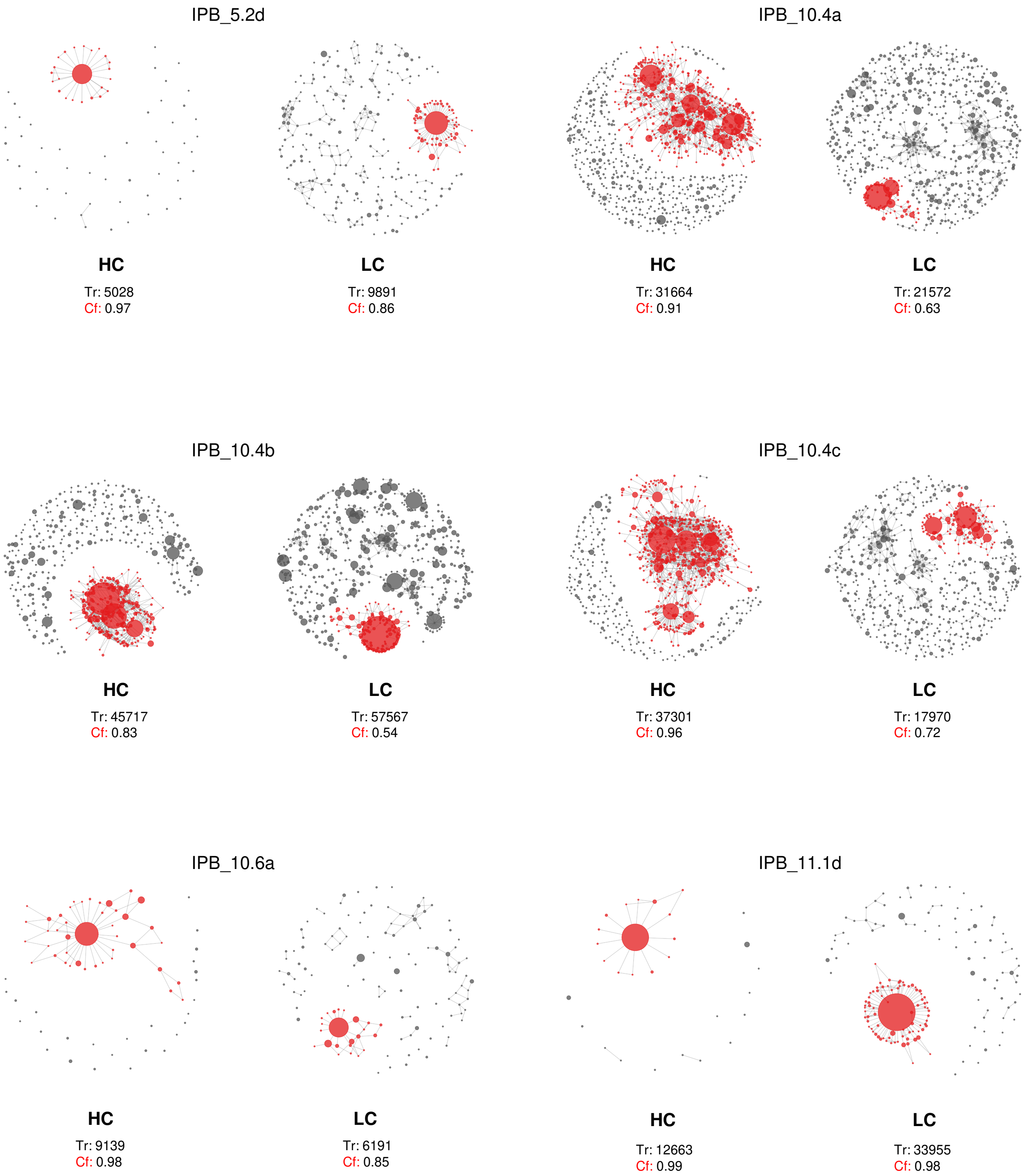

IPB_5.2d
HC
Tr: 5028
Cf: 0.97
LC
Tr: 9891
Cf: 0.86
IPB_10.4a
HC
Tr: 31664
Cf: 0.91
LC
Tr: 21572
Cf: 0.63
IPB_10.4b
HC
Tr: 45717
Cf: 0.83
LC
Tr: 57567
Cf: 0.54
IPB_10.4c
HC
Tr: 37301
Cf: 0.96
LC
Tr: 17970
Cf: 0.72
IPB_10.6a
HC
Tr: 9139
Cf: 0.98
LC
Tr: 6191
Cf: 0.85
IPB_11.1d
HC
Tr: 12663
Cf: 0.99
LC
Tr: 33955
Cf: 0.98

Supplementary Figure 5. Clonality network plots of monoclonal DLBCL samples. Clonality network plots display clonal structures of immunoglobulin heavy and light chains of monoclonal diffuse large B-cell lymphoma samples from *ITP2-M;Rosa26$^{PB/+}$;Blm$^{m3/m3}$ (IPB)* mice. Each clone (defined by a unique CDR3 sequence) constitutes a node of the clonality network. The size of the node scales with the third root of the count of the reads assigned to it. A link between two nodes was drawn if the clones mapped to identical V and J genes and differed by at most 1 bp in their CDR3 sequence. The complexity of the branching of a clone (i.e. number of subclones) is a measure for the grade of somatic hypermutation. Clones defined by a unique V(D)J rearrangement that contained more than 10% of the total reads are highlighted in color. Samples were defined as monoclonal if the main clone contained more than 40% of the total reads and the clone with the second highest read count constituted less than 10% of the total read count. HC, Heavy chain; LC; Light chain; Tr: Total reads; Cf: Fraction of clone.

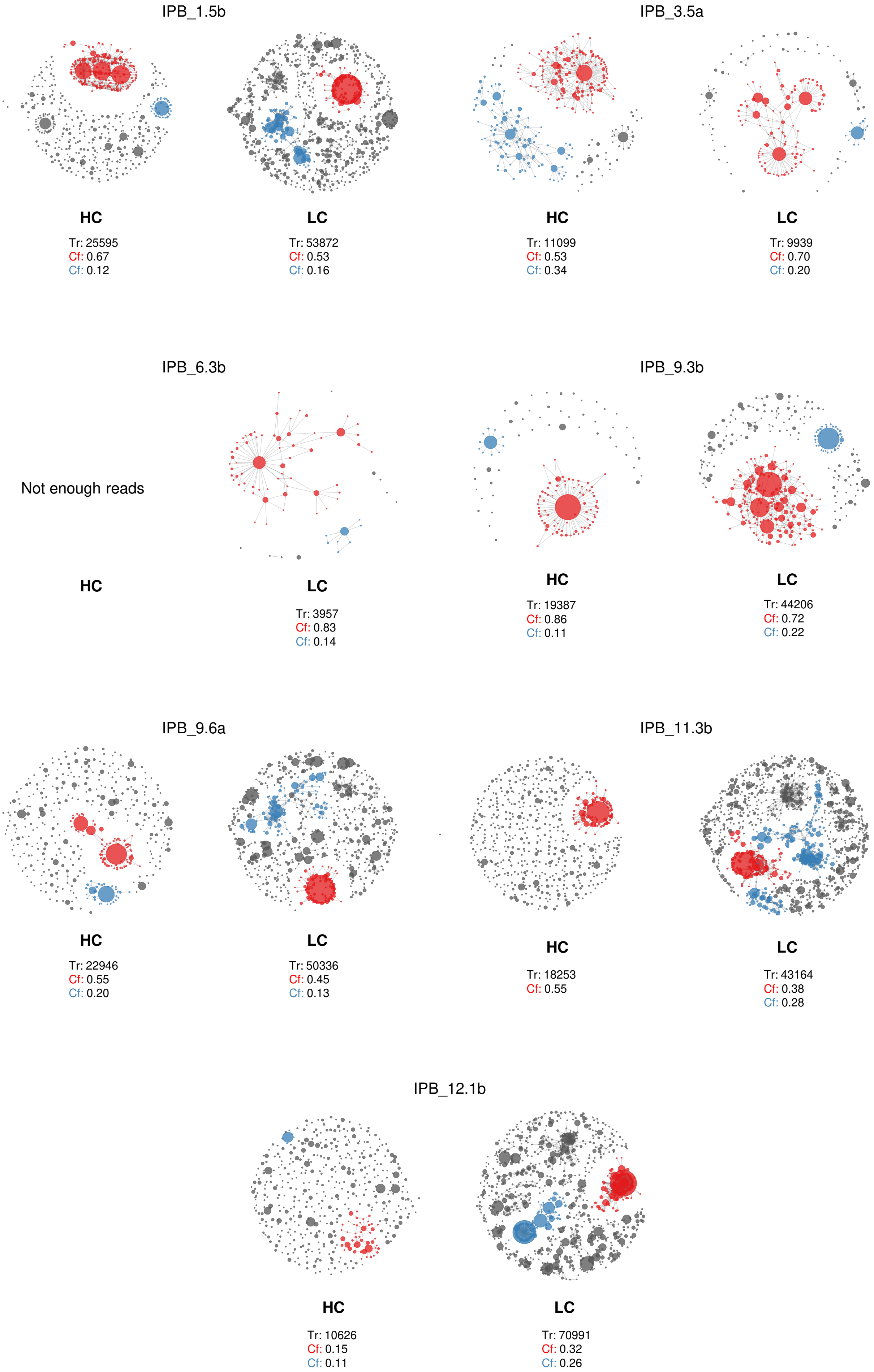
IPB_1.5b
HC
Tr: 25595
Cf: 0.67
Cf: 0.12
LC
Tr: 53872
Cf: 0.53
Cf: 0.16
IPB_3.5a
HC
Tr: 11099
Cf: 0.53
Cf: 0.34
LC
Tr: 9939
Cf: 0.70
Cf: 0.20
IPB_6.3b
Not enough reads
HC
LC
Tr: 3957
Cf: 0.83
Cf: 0.14
IPB_9.3b
HC
Tr: 19387
Cf: 0.86
Cf: 0.11
LC
Tr: 44206
Cf: 0.72
Cf: 0.22
IPB_9.6a
HC
Tr: 22946
Cf: 0.55
Cf: 0.20
LC
Tr: 50336
Cf: 0.45
Cf: 0.13
IPB_11.3b
HC
Tr: 18253
Cf: 0.55
LC
Tr: 43164
Cf: 0.38
Cf: 0.28
IPB_12.1b
HC
Tr: 10626
Cf: 0.15
Cf: 0.11
LC
Tr: 70991
Cf: 0.32
Cf: 0.26

Supplementary Figure 6*.* Clonality network plots of biclonal DLBCL samples. Clonality network plots display clonal structures of immunoglobulin heavy and light chains of biclonal diffuse large B-cell lymphoma samples from *ITP2-M;Rosa26$^{PB/+}$;Blm$^{m3/m3}$ (IPB)* mice. Each clone (defined by a unique CDR3 sequence) constitutes a node of the clonality network. The size of the node scales with the third root of the count of the reads assigned to it. A link between two nodes was drawn if the clones mapped to identical V and J genes and differed by at most 1 bp in their CDR3 sequence. The complexity of the branching of a clone (i.e. number of subclones) is a measure for the grade of somatic hypermutation. Clones defined by a unique V(D)J rearrangement that contained more than 10% of the total reads are highlighted in color. Samples were defined as biclonal if two clones, each constituting more than 10% of the total number of reads, were present. HC, Heavy chain; LC; Light chain; Tr: Total reads; Cf: Fraction of clone.

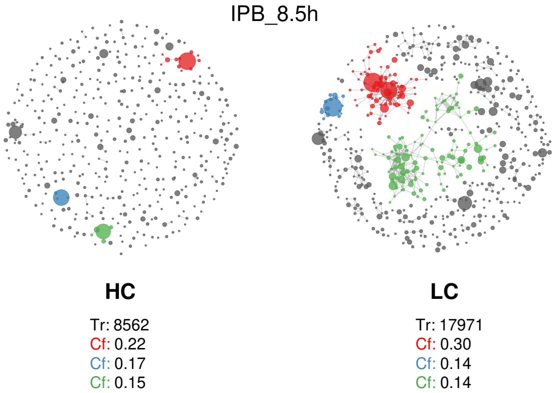

Supplementary Figure 7. Clonality network plots of a polyclonal DLBCL sample. Clonality network plots display clonal structures of immunoglobulin heavy and light chains of a polyclonal diffuse large B-cell lymphoma sample from an *ITP2-M;Rosa26$^{PB/+}$;Blm$^{m3/m3}$ (IPB)* mouse. Each clone (defined by a unique CDR3 sequence) constitutes a node of the clonality network. The size of the node scales with the third root of the count of the reads assigned to it. A link between two nodes was drawn if the clones mapped to identical V and J genes and differed by at most 1 bp in their CDR3 sequence. The complexity of the branching of a clone (i.e. number of subclones) is a measure for the grade of somatic hypermutation. Clones defined by a unique V(D)J rearrangement that contained more than 10% of the total reads are highlighted in color. Samples were defined as polyclonal if three or more clones, each constituting more than 10% of the total number of reads, were identified. HC, Heavy chain; LC; Light chain; Tr: Total reads; Cf: Fraction of clone.

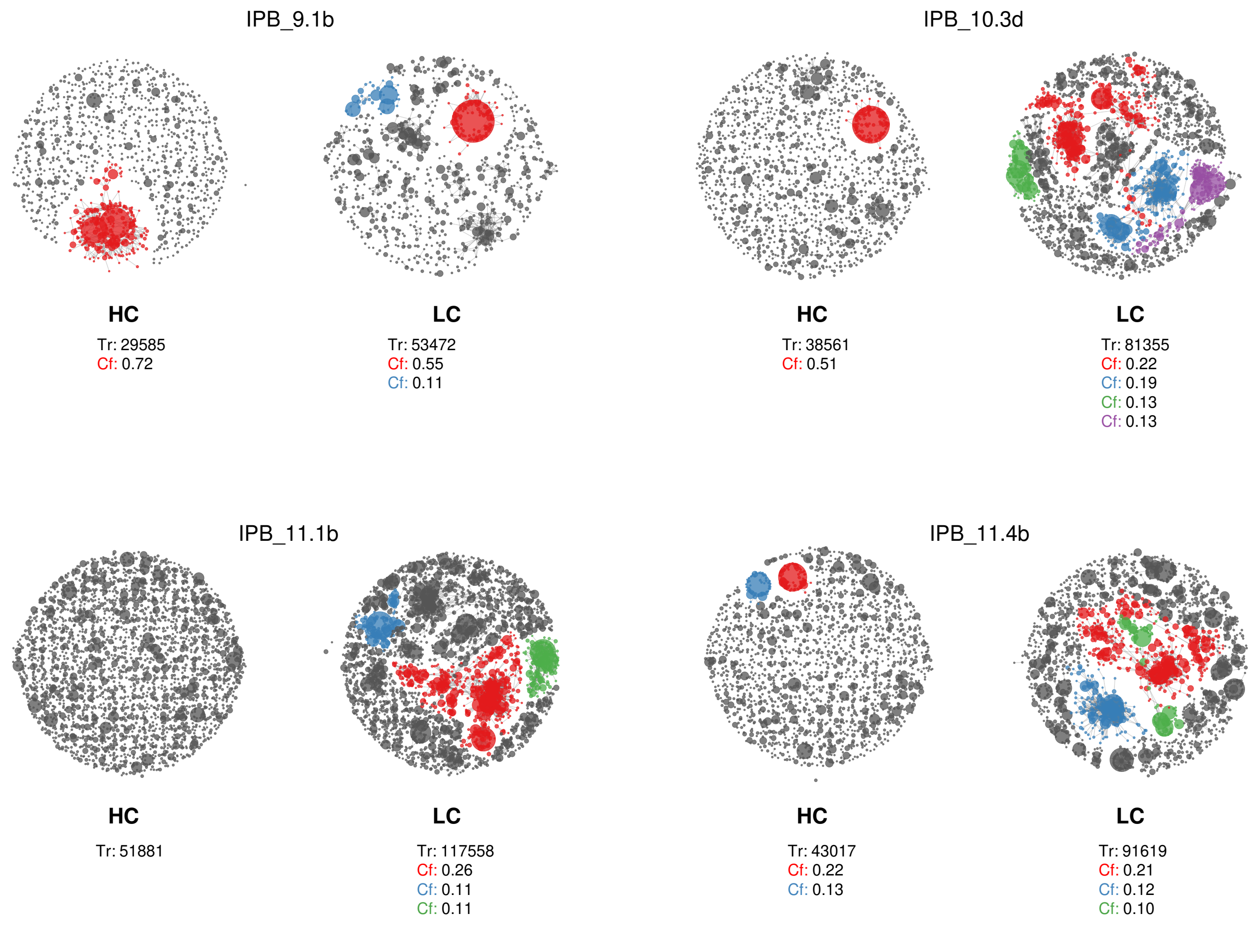

Supplementary Figure 8. Clonality network plots of samples with divergences in the clonal structure between heavy and light chain. Clonality network plots display clonal structures of immunoglobulin heavy and light chains of diffuse large B-cell lymphoma samples from *ITP2-M;Rosa26$^{PB/+}$;Blm$^{m3/m3}$ (IPB)* mice. Each clone (defined by a unique CDR3 sequence) constitutes a node of the clonality network. The size of the node scales with the third root of the count of the reads assigned to it. A link between two nodes was drawn if the clones mapped to identical V and J genes and differed by at most 1 bp in their CDR3 sequence. The complexity of the branching of a clone (i.e. number of subclones) is a measure for the grade of somatic hypermutation. Clones defined by a unique V(D)J rearrangement that contained more than 10% of the total reads are highlighted in color. Displayed are clonality network plots of samples which show divergences in the clonal structure between heavy and light chain. These samples were excluded from further analyses. HC, Heavy chain; LC; Light chain; Tr: Total reads; Cf: Fraction of clone.

A

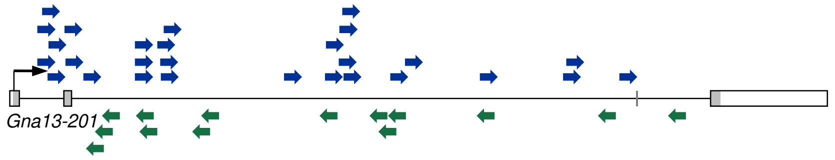

B

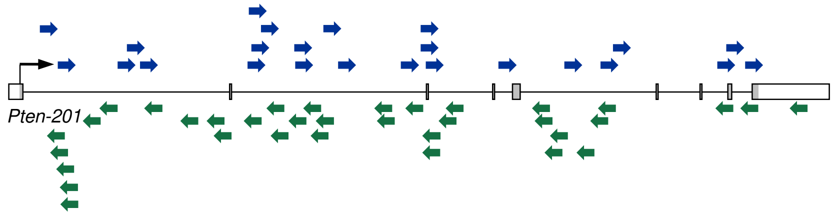

Supplementary Figure 9. Tumor suppressor genes typically show an inactivating *ITP2* insertion pattern. Transposon insertions that were identified by quantitative transposon insertion site sequencing (QiSeq) in diffuse large B-cell lymphoma (DLBCL) samples from *ITP2-M;Rosa26*$^{PB/+}$*;Blm*$^{m3/m3}$ mice overtly show patterns consistent with gene-inactivation being the cancer-promoting mechanism: *ITP2* insertions are distributed over the whole length of a gene without a bias for a specific transposon orientation (indicated by blue and green arrows). As examples, the insertion patterns of two known DLBCL tumor suppressor genes (**A**) *Gna13* and (**B**) *Pten* are shown. Each arrow represents an individual insertion. Exons are displayed by light gray boxes and 5‘ and 3‘ untranslated regions are white. The black arrow indicates the transcription start site. Illustrated are the consensus coding sequences (CCDS) of the genes. Transposon insertions with a read coverage ≥ 20 are shown.

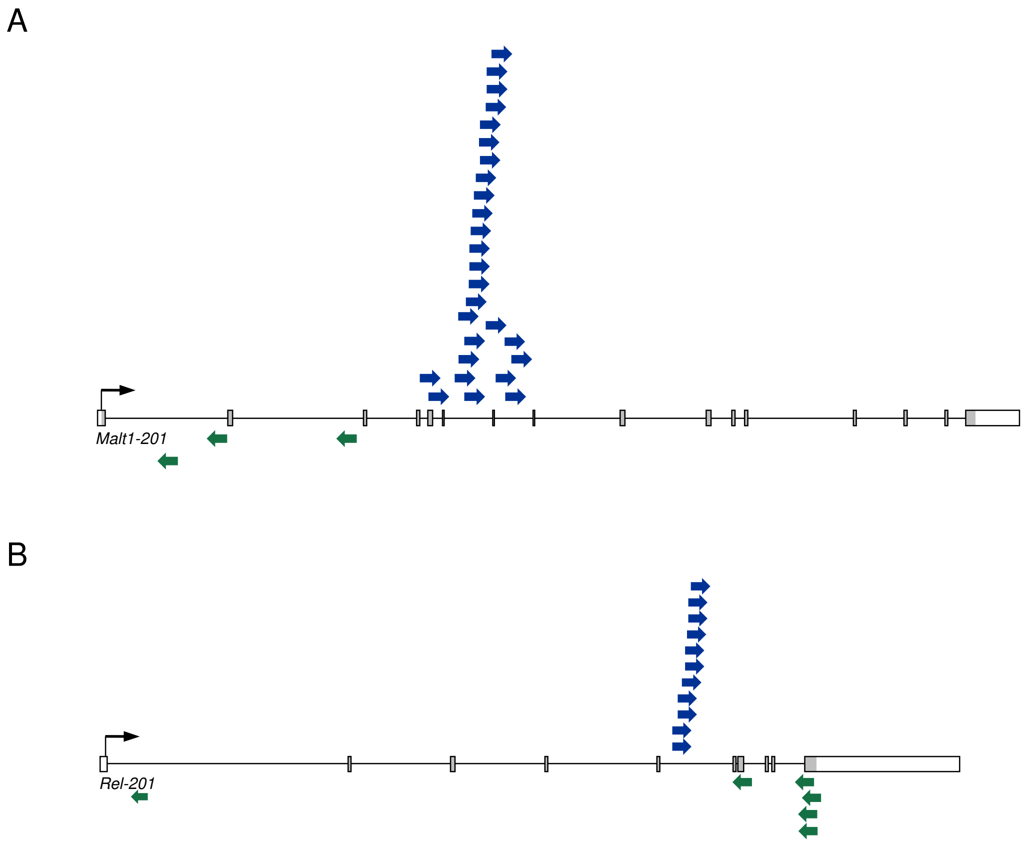

Supplementary Figure 10. Oncogenes are rarely activated by *ITP2* insertions. Transposon insertions that were identified by quantitative transposon insertion site sequencing (QiSeq) in diffuse large B-cell lymphoma (DLBCL) samples from *ITP2-M;Rosa26*$^{PB/+}$*;Blm*$^{m3/m3}$ mice typically show a gene-inactivating pattern. In rare instances, activation of oncogenes by *ITP2* insertions was observed. Transposon insertions in the DLBCL oncogenes (**A**) *Malt1* and (**B**) *Rel* are shown. *ITP2* insertions show a clear orientation bias (depicted by blue and green arrows) suggesting possible gene activation by a cryptic weak promoter element within transposon inverted terminal repeats. Additionally, in the case of *Rel*, transposon insertion within the 3‘ untranslated region might indicate interference with protein translation. Each arrow represents an individual insertion. Exons are displayed by light gray boxes and 5‘ and 3‘ untranslated regions by white ones. The black arrow indicates the transcription start site. Illustrated are the consensus coding sequences (CCDS) of the genes. Transposon insertions with a read coverage ≥ 20 are shown.

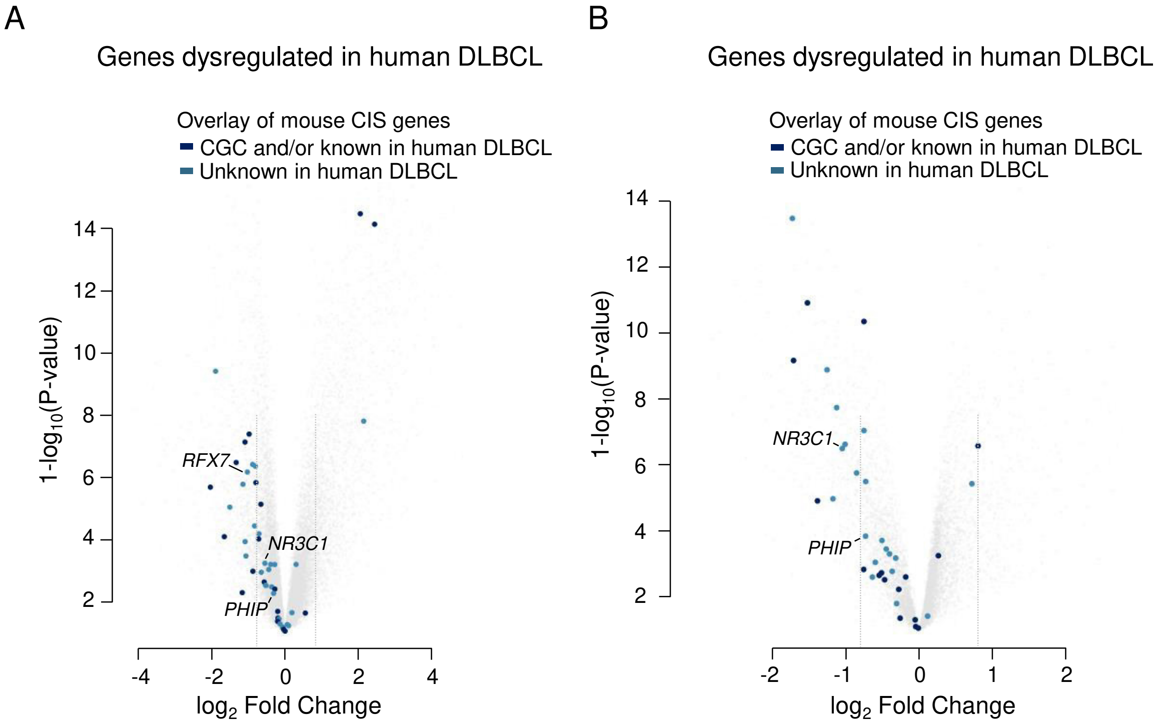

Supplementary Figure 11. Genes from the top 50 CIS list are downregulated in human B-cell lymphomas. Volcano plots show negatively (left) and positively (right) regulated genes in human diffuse large B-cell lymphoma samples relative to non-malignant B cells (centroblasts). Gray lines indicate log2 fold changes of -0.8 and 0.8. Dark blue colored points represent human orthologues of genes from the top 50 CIS list that are included in the Cancer Gene Census (CGC) database and/or have already implicated roles in diffuse large B-cell lymphoma (DLBCL). Light blue colored points depict candidate genes with unknown function in DLBCL. Data from publically available datasets (**A**) GSE12195 and (**B**) GSE2350.

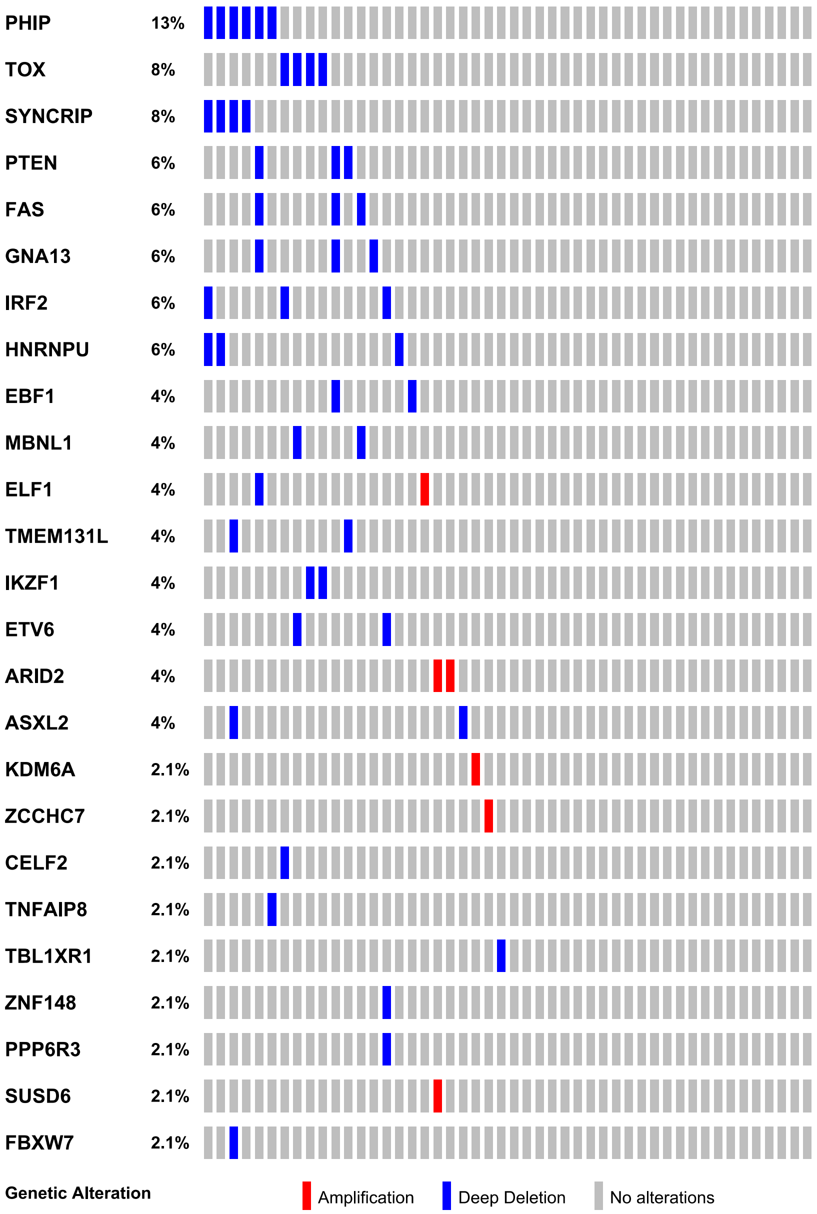

Supplementary Figure 12. Oncoplots showing copy number alterations of candidate tumor suppressor genes in human DLBCL. The existence of copy number alterations (CNA) in human orthologues of the 50 top CIS genes in our screen was interrogated in the TCGA-DLBC dataset (Pan-Cancer Atlas from Cancer Genome Atlas (TCGA) consortium). Featured are genes with CNAs, as estimated by GISTIC 2.0 analysis. Source and output from cBioPortal (http://www.cbioportal.org/). The two genes with oncogenic insertions in our screen (*Malt1* and *Rel*) were not included in the analysis. Note that heterozygous deletions are not shown.

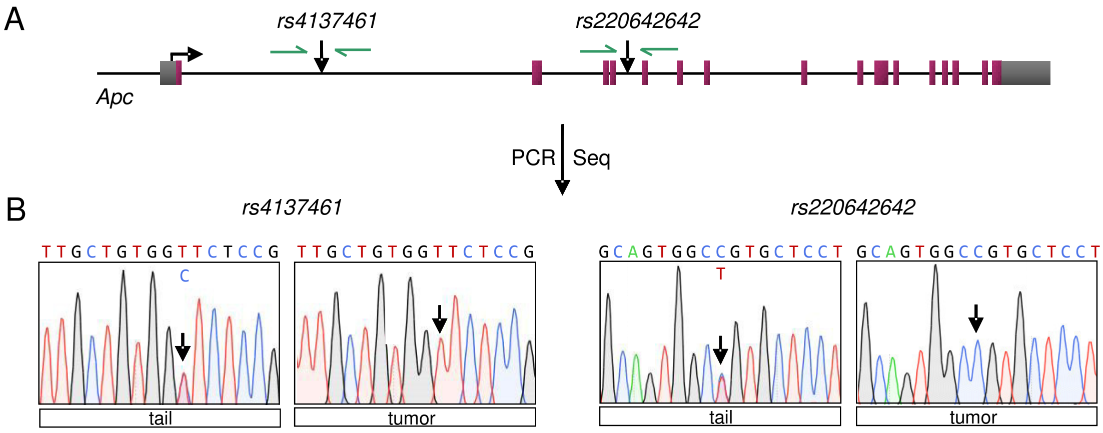

Supplementary Figure 13. Analysis of loss of heterozygosity in an *IPB* tumor cell line. For loss of heterozygosity (LOH) analysis, two single nucleotide polymorphism (SNP) containing regions within *Apc* were amplified and sequenced in DNA from an small intestine tumor cell line (harboring a high coverage *ITP2* insertion in *Apc*) and tail of an *ITP2-M;Rosa26*$^{PB/+}$*;Blm*$^{m3/m3}$ *(IPB)* mouse. (**A**) Structure of the mouse *Apc* gene with indicated positions of SNPs *rs4137461* and *rs220642642* (black arrows) and PCR primers (green arrows). (**B**) Sequencing results of tumor cell line and tail DNA for each SNP show that LOH occurs in the tumor. Seq., sequencing.

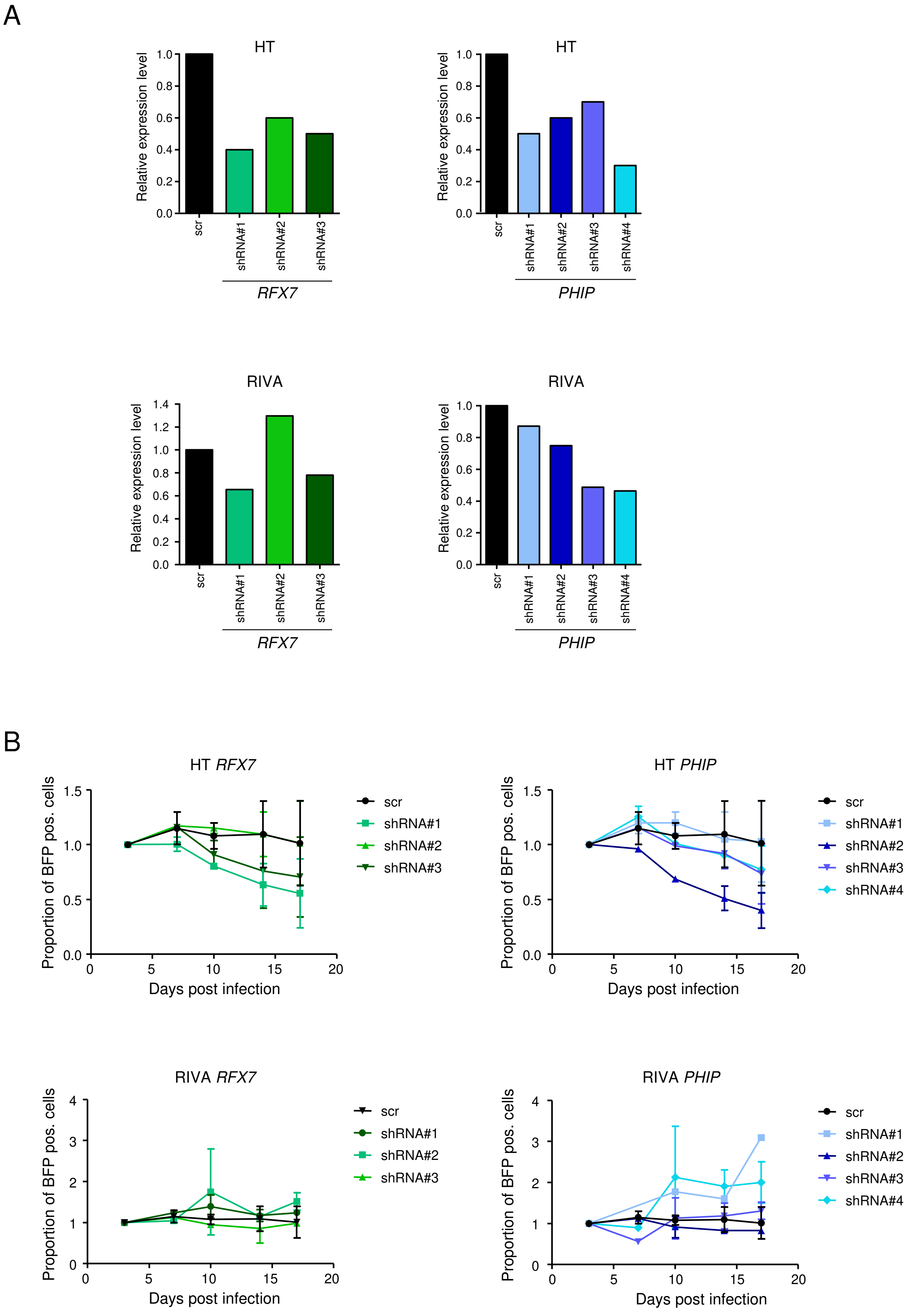
A
HT
Relative expression level
scr
shRNA#1
shRNA#2
shRNA#3
RFX7
HT
scr
shRNA#1
shRNA#2
shRNA#3
shRNA#4
PHIP
RIVA
Relative expression level
RFX7
RIVA
PHIP
B
HT RFX7
Proportion of BFP pos. cells
Days post infection
scr
shRNA#1
shRNA#2
shRNA#3
HT PHIP
scr
shRNA#1
shRNA#2
shRNA#3
shRNA#4
RIVA RFX7
RIVA PHIP

Supplementary Figure 14. Functional cell culture based competition assays for *in vitro* analysis of gene function. Cell culture based competition assays were performed to analyze if knockdown of the candidate genes *RFX7* and *PHIP* influences cell proliferation *in vitro*. For this, the human GCB DLBCL cell line HT and the human ABC DLBCL cell line RIVA were transduced with lentiviral vectors expressing blue fluorescent protein (BFP) and either a scrambled shRNA control (scr) or shRNAs targeting *RFX7* (3 shRNAs) or *PHIP* (4 shRNAs). (**A**) For analysis of knockdown efficiencies, BFP positive HT and RIVA cells were sorted 17 days post infection. *RFX7* and *PHIP* expression was determined by real time quantitative PCR (qPCR) using primers specific for *RFX7* and *PHIP* transcripts. For normalization of RNA input, GAPDH qPCR was performed. Transduced cells (with the exception of RIVA cells targeted with RFX7 shRNA#2) showed a knockdown in *RFX7* or *PHIP* expression. One out of two replicates is shown. (**B**) For competition assays, transduced BFP positive cells were co-cultured with non-transduced cells and the proportion of BFP positive cells was analyzed on day 3, 7, 10, 14 and 17 post infection. Percentage of BFP positive cells was normalized to day 3 post transduction. Data was analyzed with a 2-way ANOVA allowing for interaction effects. No significant time specific effects between conditions (*RFX7* knockdown, *PHIP* knockdown and scr cells) were detected for all experiments. Line charts show the course of the proportion of BFP positive cells over 14 days. Data represented as mean of two replicates ± standard error of the mean. Pos, positive.

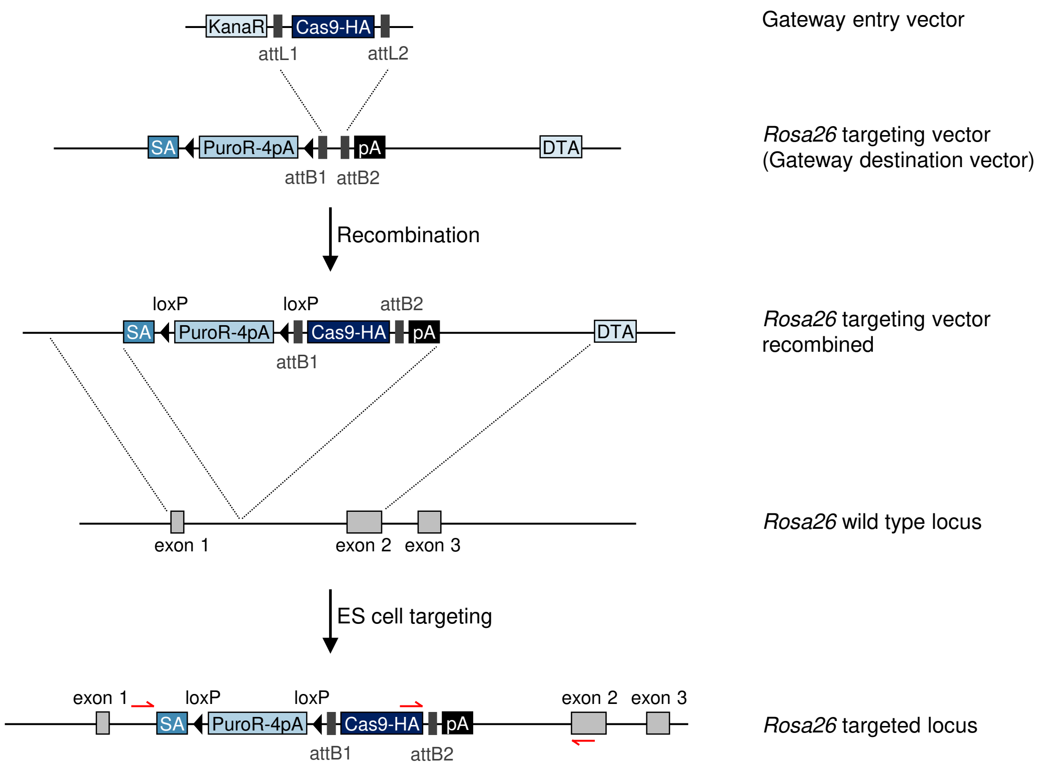

Supplementary Figure 15. Alleles and vectors for generation of the *Rosa26*^{Cas9} knock-in mouse line. To generate knock-in mice expressing Cas9 from the endogenous *Rosa26* promoter, we first cloned a human codon-optimized hemagglutinin (HA)-tagged Cas9 (Cas9-HA) sequence derived from *Streptococcus pyogenes* into a Gateway-compatible entry vector. We then shuttled the Cas9-HA sequence into the *Rosa26*-targeting Gateway destination vector with a loxP-flanked puromycin-resistance-containing stop cassette. Embryonic stem (ES) cell (JM8) targeting, blastocyst injections and subsequent breeding steps were performed using standard protocols/techniques. Upon generation of conditional *Rosa26*$^{LSL\text{-}Cas9}$ knock-in mice, we derived a constitutive *Rosa26*^{Cas9} mouse line by deletion of the loxP-flanked stop cassette in the germline using *Tg*$^{Actb\text{-}Cre}$ mice. *Rosa26*$^{LSL\text{-}Cas9}$ and *Rosa26*^{Cas9} mice were established and maintained on a pure *C57BL/6* background. Genotyping primers are indicated by red arrows. KanaR, kanamycin resistance; attL1/attL2, Gateway recombination sites; SA, splice acceptor; PuroR, puromycin resistance; attB1/attB2, Gateway recombination sites; pA, polyadenylation signal; DTA, diphtheria toxin alpha.

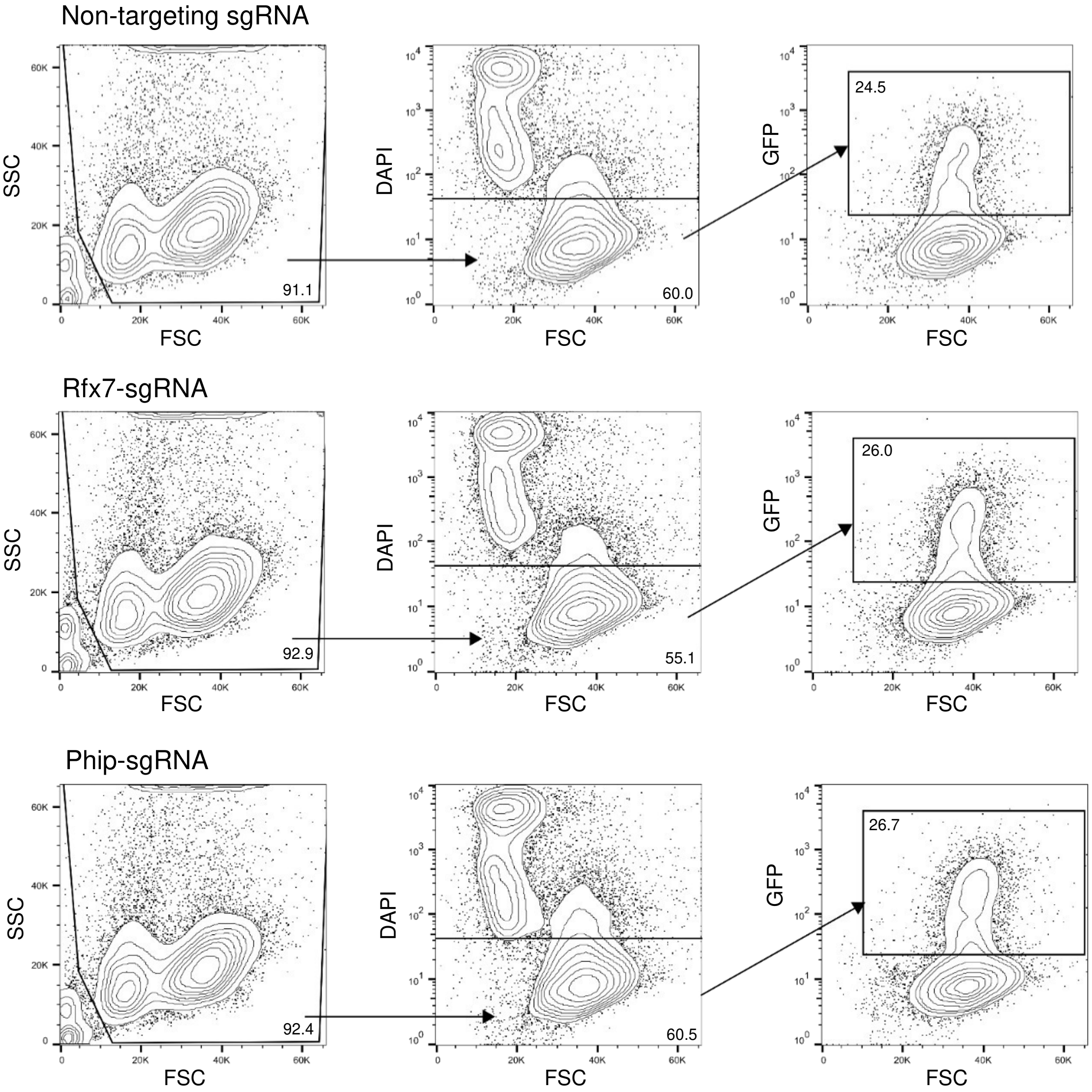

Supplementary Figure 16. Infection of fetal liver cells for CRISPR/Cas9-based *in vivo* validation model. Representative contour plots from flow cytometry analysis of fetal liver cells infected with GFP-tagged lentiviral single guide RNA (sgRNA) vectors. Left plots: Forward scatter (FSC; x axis) and side scatter (SSC; y axis) measurements; gating to exclude cell debris. Middle plots: DAPI fluorescence on y axis; gating to exclude dead cells. Right plots: GFP fluorescence on y axis; gating to quantify GFP positive viable cells.

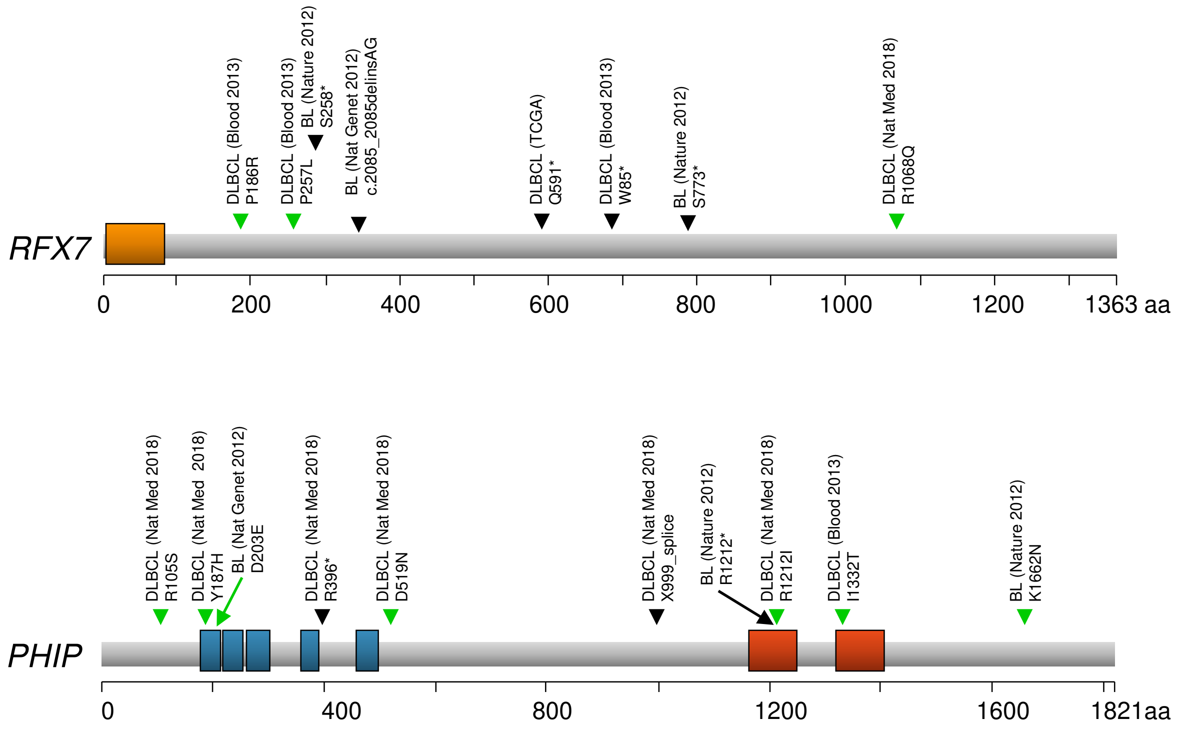

Supplementary Figure 17. ***RFX7* and *PHIP* mutations in human B-cell lymphoma.** Schemes display location of mutations (single nucleotide variations and small insertions/deletions) within *RFX7* (above) and *PHIP* (below) in human diffuse large B-cell lymphoma (DLBCL) and Burkitt lymphoma (BL). Truncating mutations are displayed by black arrow heads, missense mutations by green arrow heads. Mutation data was sourced from the following studies: TCGA diffuse large B-cell lymphoma (DLBC) dataset (TCGA Research Network: http://cancergenome.nih.gov), Schmitz *et al.* Nature 2012, Morin *et al.* Blood 2013, Richter *et al.* Nature Genetics 2012, Chapuy *et al.* Nature Medicine 2018. Indicated functional protein domains: PHIP: WD40 domain (blue) and bromodomain (red), RFX7: RFX DNA-binding domain (orange).

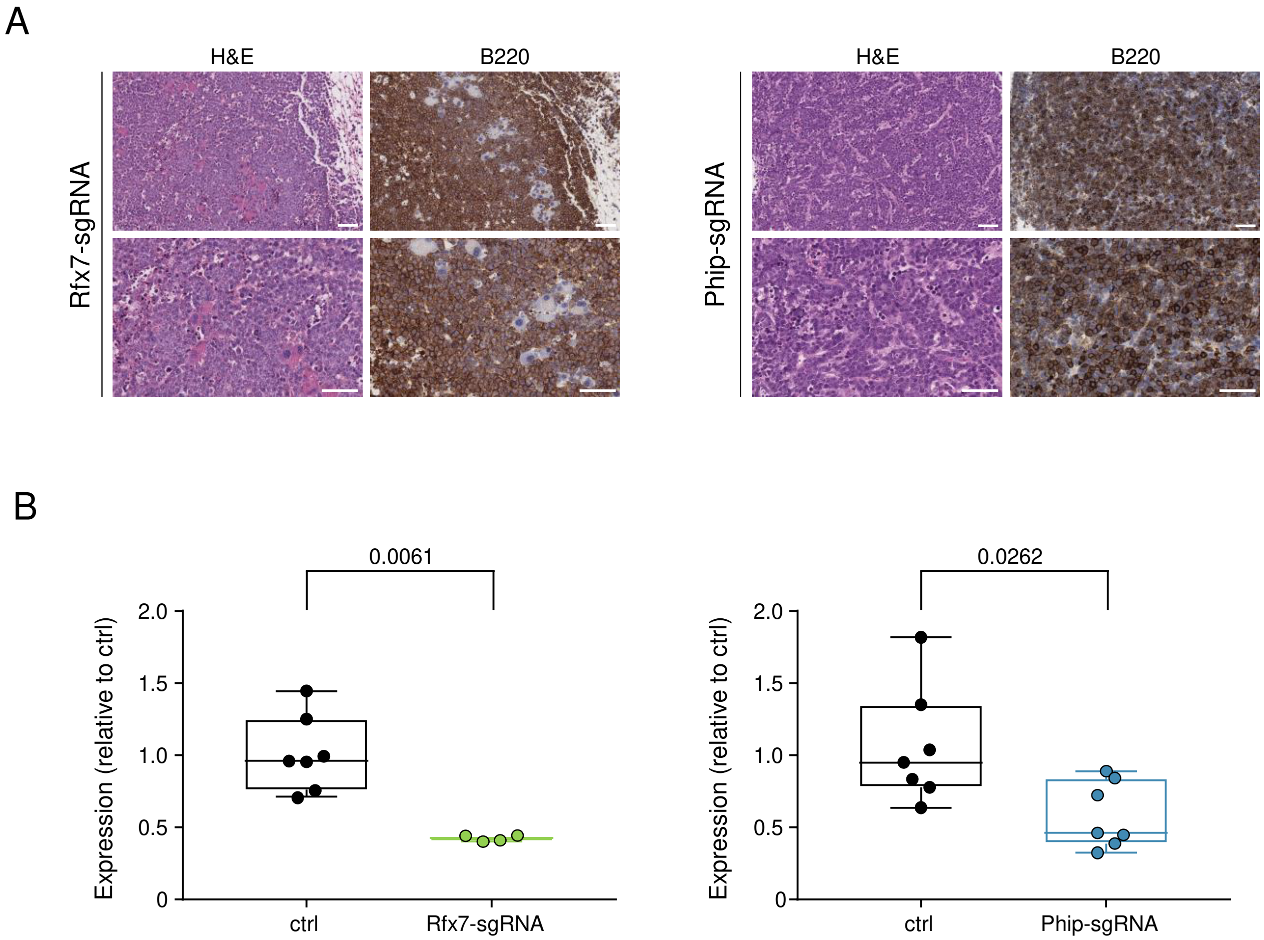

Supplementary Figure 18. Analysis of tumors from the CRISPR/Cas9-based *in vivo* validation model. (**A**) Representative microscopic images of tumors from mice reconstituted with Rfx7-sgRNA (left) and Phip-sgRNA (right) *Eμ-myc;Rosa26*^{Cas9} HSPCs. Mice developed B-cell lymphomas exhibiting strong B220/CD45R positivity (right panels). Scale bars: 50 µm. H&E, Hematoxylin and eosin staining. (**B**) Box plots showing Rfx7 (left) and Phip (right) expression analyzed by real time quantitative PCR (qPCR) in tumors from mice reconstituted with Rfx7-sgRNA (n = 4) and Phip-sgRNA (n = 7) *Eμ-myc;Rosa26*^{Cas9} HSPCs, respectively. Expression in tumors from the Rfx7- and Phip-sgRNA cohorts was compared to expression levels in endogenous tumors (n = 7) from *Eμ-myc* mice. For normalization of RNA input, Gapdh qPCR was performed. Whiskers: minimum to maximum; horizontal line: median. A Mann-Whitney test was performed to test the significance of change in expression. Ctrl, controls.

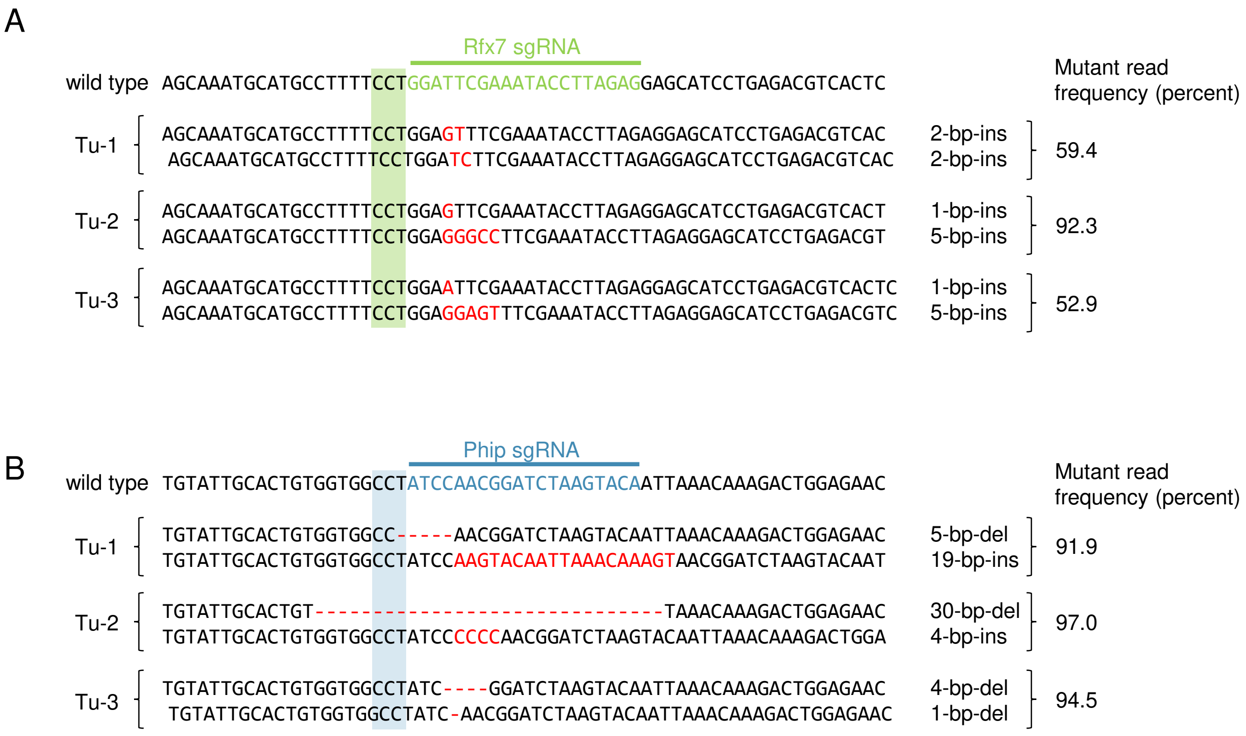

Supplementary Figure 19. Amplicon-based next-generation sequencing for indel identification at target sites. For detection of insertions/deletions (indels) in tumors derived from the CRISPR/Cas9-based *in vivo* validation approach, amplicon-based next-generation sequencing of the target regions followed by indel analysis was performed. For all analyzed samples (n = 3 tumors per gene), the presence of two clonal indels was identified at the predicted Cas9 cleavage site. Mutant read frequencies represent the fraction of mutant-reads/all-reads at individual target sites. (**A**) At the *Rfx7* target region, small insertions (1 bp to 5 bp) were present while (**B**) at the *Phip* target site, indels ranged from 1 bp to 30 bp. Indels are indicated by red characters and the protospacer adjacent motifs next to the single guide RNAs are shaded in green and blue. Del, deletion; ins, insertion; sgRNA, single guide RNA.

Mouse ID	Cohort	Diagnosis
PB_1.3b	$Rosa26^{PB/+};Blm^{m3/m3}$	Diffuse large B-cell lymphoma
PB_1.4g	$Rosa26^{PB/+};Blm^{m3/m3}$	B-cell lymphoma
PB_1.6e	$Rosa26^{PB/+};Blm^{m3/m3}$	T-cell lymphoblastic lymphoma
PB_1.7b	$Rosa26^{PB/+};Blm^{m3/m3}$	Lymphoma
PB_2.4c	$Rosa26^{PB/+};Blm^{m3/m3}$	Diffuse large B-cell lymphoma
PB_3.2c	$Rosa26^{PB/+};Blm^{m3/m3}$	Diffuse large B-cell lymphoma
PB_3.3a	$Rosa26^{PB/+};Blm^{m3/m3}$	Diffuse large B-cell lymphoma
PB_3.3b	$Rosa26^{PB/+};Blm^{m3/m3}$	Diffuse large B-cell lymphoma
PB_3.3d	$Rosa26^{PB/+};Blm^{m3/m3}$	Diffuse large B-cell lymphoma
PB_3.3e	$Rosa26^{PB/+};Blm^{m3/m3}$	Diffuse large B-cell lymphoma with plasmacytic differentiation
PB_3.4f	$Rosa26^{PB/+};Blm^{m3/m3}$	Invasive adenocarcinoma of the uterus, low grade dysplasia in the small intestine
PB_3.4g	$Rosa26^{PB/+};Blm^{m3/m3}$	Diffuse large B-cell lymphoma
PB_3.5b	$Rosa26^{PB/+};Blm^{m3/m3}$	Low grade dysplasia in the small intestine
PB_3.6e	$Rosa26^{PB/+};Blm^{m3/m3}$	Lymphoma, Invasive adenocarcinoma of the uterus
PB_3.6g	$Rosa26^{PB/+};Blm^{m3/m3}$	Uterus sarcoma
PB_4.5e	$Rosa26^{PB/+};Blm^{m3/m3}$	Lymphoma
PB_4.5f	$Rosa26^{PB/+};Blm^{m3/m3}$	B-cell lymphoma
PB_4.5h	$Rosa26^{PB/+};Blm^{m3/m3}$	Squamous cell carcinoma of the skin
PB_4.6e	$Rosa26^{PB/+};Blm^{m3/m3}$	Diffuse large B-cell lymphoma and histiocytic sarcoma
PB_5.1d	$Rosa26^{PB/+};Blm^{m3/m3}$	Hepatocellular carcinoma
PB_5.1e	$Rosa26^{PB/+};Blm^{m3/m3}$	Diffuse large B-cell lymphoma
PB_5.2c	$Rosa26^{PB/+};Blm^{m3/m3}$	Diffuse large B-cell lymphoma with plasmacytic differentiation and histiocytic sarcoma
PB_5.2g	$Rosa26^{PB/+};Blm^{m3/m3}$	Lymphoma
PB_5.4e	$Rosa26^{PB/+};Blm^{m3/m3}$	Lymphoma
PB_5.7a	$Rosa26^{PB/+};Blm^{m3/m3}$	Leukemia
PB_5.7b	$Rosa26^{PB/+};Blm^{m3/m3}$	Papillary adenocarcinoma of the lung
PB_5.7d	$Rosa26^{PB/+};Blm^{m3/m3}$	Lymphoma
PB_6.2f	$Rosa26^{PB/+};Blm^{m3/m3}$	Diffuse large B-cell lymphoma with plasmacytic differentiation
PB_6.2g	$Rosa26^{PB/+};Blm^{m3/m3}$	Lymphoma
PB_6.2h	$Rosa26^{PB/+};Blm^{m3/m3}$	Lymphoma
PB_6.3h	$Rosa26^{PB/+};Blm^{m3/m3}$	Diffuse large B-cell lymphoma
PB_6.3i	$Rosa26^{PB/+};Blm^{m3/m3}$	Lymphoma
PB_6.3j	$Rosa26^{PB/+};Blm^{m3/m3}$	Lymphoma
PB_6.4i	$Rosa26^{PB/+};Blm^{m3/m3}$	Lymphoma
PB_7.1c	$Rosa26^{PB/+};Blm^{m3/m3}$	Diffuse large B-cell lymphoma and B-cell lymphoblastic lymphoma
PB_7.1d	$Rosa26^{PB/+};Blm^{m3/m3}$	Diffuse large B-cell lymphoma
PB_7.2c	$Rosa26^{PB/+};Blm^{m3/m3}$	Lymphoma
PB_7.3c	$Rosa26^{PB/+};Blm^{m3/m3}$	Invasive adenocarcinoma of the uterus
PB_8.3h	$Rosa26^{PB/+};Blm^{m3/m3}$	Lymphoma
PB_8.3i	$Rosa26^{PB/+};Blm^{m3/m3}$	Histiocytic sarcoma
PB_8.5f	$Rosa26^{PB/+};Blm^{m3/m3}$	Uterus sarcoma
IB_10.1a	$ITP2\text{-}M;Blm^{m3/m3}$	Lymphoma
IB_10.7e	$ITP2\text{-}M;Blm^{m3/m3}$	Lymphoma
IB_10.7i	$ITP2\text{-}M;Blm^{m3/m3}$	Lymphoma
IB_10.7g	$ITP2\text{-}M;Blm^{m3/m3}$	Lymphoma

Supplementary Table 1. $Rosa26^{PB/+};Blm^{m3/m3}$ and $ITP2\text{-}M;Blm^{m3/m3}$ control mice with tumors. Overview of $Rosa26^{PB/+};Blm^{m3/m3}$ and $ITP2\text{-}M;Blm^{m3/m3}$ control mice displaying hematopoietic and solid tumors.

Mouse ID	Solid Tumor Diagnosis
IPB_1.2d	Cervical mass
IPB_1.4e	Adenocarcinoma of the uterus
IPB_1.5a	Squamous cell carcinoma; Dysplastic polyp in small intestine
IPB_1.5b	Bronchioalveolar adenocarcinoma
IPB_1.5f	Astrocytoma
IPB_1.6d	Poorly differentiated tumor in the neck
IPB_1.7d	Pituitary adenoma; Lung adenoma
IPB_2.4b	Pituitary adenoma
IPB_2.4e	Papillary lung adenoma
IPB_2.6e	Pituitary adenoma; Hamartoma in uterus
IPB_4.1d	Adenosquamous carcinoma of the uterus
IPB_4.5g	Squamous cell carcinoma; Mixed adenocarcinoma and squamous cell carcinoma of the stomach
IPB_4.6d	Papillary lung adenoma
IPB_5.5e	Pituitary adenoma
IPB_6.2f	Bronchoalveolar adenocarcinoma; Invasive adenocarcinoma of the uterus
IPB_8.1c	Stomach adenocarcinoma
IPB_9.2d	Skin appendage tumor
IPB_9.2g	Papillary adenomatous polyp of the uterus; Pituitary adenoma
IPB_9.6a	Cystic endometrial hyperplasia; Squamous cell metaplasia plus dysplasia of the transitional epithelium of the urinary bladder
IPB_9.7d	Adenomatous polyp of the uterus
IPB_10.4d	Astrocytoma
IPB_10.4e	Squamous cell carcinoma
IPB_10.6b	Pituitary adenoma
IPB_11.1c	Pituitary adenoma
IPB_11.1d	Lung adenoma
IPB_11.5b	Prostate dysplasia
IPB_12.1b	Pituitary adenoma
IPB_12.3d	Hepatocellular carcinoma

Supplementary Table 2. *IPB* mice with solid tumors. Overview of all *ITP2-M;Rosa26*$^{PB/+}$*;Blm*$^{m3/m3}$ *(IPB)* mice developing solid tumors that were characterized histopathologically.

Gene A	Gene B	P-value	Adjusted P-value	Odds Ratio	LCL	UCL
Fas	*Gna13*	0.0008	0.02915	22.48	2.58	1099.87
Ptprc	*Mef2c*	0.0017	0.04666	17.75	2.09	855.32
Ebf1	*Gna13*	0.0011	0.03651	10.64	2.08	76.64
Ebf1	*Tmem131l*	0.0002	0.00880	1.00	3.4	Inf
Tox	*Basp1*	0.0012	0.03724	12.45	2.15	136.86
Tox	*Erp44*	0.0004	0.01638	22.25	2.66	1064.94
Tox	*Pds5a*	0.0012	0.03651	1.00	2.41	Inf
Zbtb20	*Nf1*	0.0016	0.04595	9.91	2.11	59.16
Zbtb20	*Pds5a*	0.0002	0.00792	1.00	4.03	Inf
Zbtb20	*Elf1*	0.0005	0.02041	12.54	2.58	78.83
Mef2c	*Elf1*	0.0016	0.04636	9.52	2.06	54.06
Nf1	*Etv6*	0.0004	0.01814	12.27	2.51	77.42
Basp1	*Tmem131l*	0.0008	0.02915	12.90	2.46	95.48
Basp1	*Hnrnpa2b1*	0.0014	0.04189	12.77	2.1	144.8
Basp1	*Chd2*	0.0008	0.02915	21.18	2.38	1043.61
Phip	*Arid2*	0.0008	0.02915	24.39	2.52	1249.42
Nr3c1	*Chd2*	0.0008	0.02915	15.78	2.38	189.46
Rasa2	*Pds5a*	0.0012	0.03651	12.69	2.16	102.21
Rasa2	*Hnrnpu*	0.0003	0.01460	1.00	3.64	Inf
Inpp5d	*Elf1*	0.0012	0.03651	19.50	2.25	947.94

Supplementary Table 3. Co-occurrence analysis of the top 50 CIS genes. A Fisher's exact test was performed for co-occurrence interference of the top 50 CIS genes. Listed are the 20 unique pairs that remained significant after multiple testing correction (Benjamini-Hochberg). Associated odds ratios with the corresponding 95% confidence intervals are also shown. LCL, lower confidence limit; UCL, upper confidence limit; inf, infinite.

Survival	Gene Name	P-value	Adjusted P-value	Hazard Ratio	LCL	UCL	# Patients with LE (censored/total)	# Patients with HE (censored/total)
OS	*ZCCHC7*	0.00005	0.0055	0.56	0.396	0.792	110/212	160/212
OS	*TOX*	0.00006	0.006	0.562	0.398	0.792	112/212	158/212
OS	*NR3C1*	0.0001	0.00803	0.55	0.392	0.772	106/212	164/212
OS	*RFX7*	0.00015	0.00964	0.501	0.356	0.705	107/212	163/212
OS	*MEF2C*	0.00017	0.01028	0.583	0.419	0.812	118/212	152/212
OS	*EBF1*	0.00019	0.01087	0.556	0.399	0.777	125/212	145/212
OS	*GNA13*	0.00021	0.01105	0.58	0.413	0.815	112/212	158/212
OS	*PPP6R3*	0.00084	0.02356	0.541	0.388	0.755	124/212	176/212
OS	*MBNL1*	0.00173	0.03502	0.563	0.402	0.789	106/212	164/212
OS	*ZBTB20*	0.00182	0.03579	0.647	0.466	0.897	133/212	137/212
OS	*ERP44*	0.00279	0.04416	0.619	0.444	0.862	111/212	159/212
PFS	*ZCCHC7*	0.00001	0.00379	0.536	0.382	0.752	111/212	156/212
PFS	*TOX*	0.00013	0.01535	0.599	0.429	0.836	113/212	154/212
PFS	*MEF2C*	0.0002	0.01998	0.606	0.436	0.841	119/212	148/212
PFS	*EBF1*	0.00022	0.02076	0.576	0.416	0.796	123/212	144/212
PFS	*RFX7*	0.0011	0.04538	0.559	0.405	0.773	115/212	152/212
PFS	*NR3C1*	0.00111	0.04541	0.624	0.451	0.864	111/212	156/212
PFS	*GNA13*	0.00148	0.05295	0.649	0.468	0.899	111/212	156/212
PFS	*ERP44*	0.00189	0.05859	0.618	0.448	0.854	115/212	152/212
PFS	*PPP6R3*	0.0022	0.0621	0.578	0.419	0.798	128/212	164/212
PFS	*MBNL1*	0.00276	0.06895	0.585	0.423	0.809	110/212	157/212
PFS	*ZBTB20*	0.01142	0.13193	0.73	0.528	1.007	133/212	137/212

Supplementary Table 4. Clinical relevance of CIS genes in human DLBCL. Human orthologues of genes from the top 50 CIS list (with tumor suppressive function; *MALT1* and *REL* excluded) for which low expression significantly correlates with poor overall survival (OS) in a human diffuse large B-cell lymphoma dataset (GSE31312 [*n*=424 patients]) are listed. For these genes, data for association with progression-free survival (PFS) are also shown. For each gene, the cohort was stratified into "low" (LE; below median expression) or "high" expression (HE, above median). LCL, lower confidence limit; UCL, upper confidence limit; #, number.
