# Supplementary material for: PiggyBac transposon tools for recessive screening identify B-cell lymphoma drivers in mice
Source: Nat Commun. 2019 Mar 29;10:1415. doi: 10.1038/s41467-019-09180-3 (PMC6440946; doi:10.1038/s41467-019-09180-3)
Supplement: Supplementary file 2 — Description of Additional Supplementary Files [file 41467_2019_9180_MOESM2_ESM.docx]

**Description of Additional Supplementary Files**

File Name: Supplementary Data 1

Description: Numbers of triple transgenic offspring in ITP breedings. Overview of numbers of triple transgenic mice born alive in breedings for generation of *ITP1-C;Rosa26^PB/+^;Blm^m3/m3^* and *ITP2-M;Rosa26^PB/+^;Blm^m3/m3^* mice. Matings were usually performed as trios (two female mice and one male mouse).

File Name: Supplementary Data 2

Description: *IPB* mice with hematopoietic tumors. Overview of all *ITP2-M;Rosa26^PB/+^;Blm^m3/m3^ (IPB)* mice showing hematopoietic cancers that were characterized histopathologically. Infiltrate of other hematopoietic tumor listed in brackets. F, female; m, male; QiSeq, quantitative transposon insertion site sequencing.

File Name: Supplementary Data 3

Description: Immune repertoire analysis of tumors from IPB mice. Results from B-cell receptor repertoire sequencing of immunoglobulin heavy and light chains of 30 diffuse large B-cell lymphomas from *ITP2-M;Rosa26^PB/+^;Blm^m3/m3^* mice are shown.

File Name: Supplementary Data 4

Description: Common insertion sites resulting from TAPDANCE analysis. List of common insertion sites (CIS) identified by TAPDANCE (Transposon Annotation Poisson Distribution Association Network Connectivity Environment) analysis using non-redundant insertions with a read coverage ≥ 2 (n=298,439) from 42 *ITP2-M;Rosa26^PB/+^;Blm^m3/m3^* mice as an input. Only CISs identified in at least 10% of samples are listed. #, number.

File Name: Supplementary Data 5

Description: Common insertion sites resulting from CIMPL analysis. List of common insertion sites (CIS) identified by CIMPL (Common Insertion site Mapping PLatform) analysis using non-redundant insertions with a read coverage ≥ 20 (n=43,474) from 42 *ITP2-M;Rosa26^PB/+^;Blm^m3/m3^* mice as an input. Only CISs identified across all analyzed scales (30,000, 50,000, 70,000 and 90,000) are listed. Chr, chromosome; #, number.

File Name: Supplementary Data 6

Description: Pathway enrichment analysis using the top 50 CIS genes. Overview of enriched molecular signatures analyzed using the Reactome gene sets from the MSigDB database v6.2 (Broad Institute, Inc., Massachusetts Institute of Technology and Regents of the University of California) and the top 50 CIS genes as an input. Gene sets with an FDR q-value <0.05 are listed. FDR, false discovery rate.

File Name: Supplementary Data 7

Description: Potentially druggable genes from the top 50 CIS genes. Overview of the output from the Drug Gene Interaction Database (DGIdb; http://www.dgidb.org/) that was mined to identify potentially druggable genes among the top 50 CIS genes. FDA, United States Food and Drug Administration. *

File Name: Supplementary Data 8

Description: Genotyping primers.

File Name: Supplementary Data 9

Description: sgRNA oligonucleotides, shRNA oligonucleotides and PCR primers.

File Name: Supplementary Data 10

Description: Adapter and primers for B-cell receptor repertoire sequencing.
