# Supplementary material for: PiggyBac transposon tools for recessive screening identify B-cell lymphoma drivers in mice
Source: Nat Commun. 2019 Mar 29;10:1415. doi: 10.1038/s41467-019-09180-3 (PMC6440946; doi:10.1038/s41467-019-09180-3)
Supplement: Supplementary file 14 — Reporting Summary [file 41467_2019_9180_MOESM14_ESM.pdf]

nature research

Corresponding author(s): Roland Rad

Last updated by author(s): Jan 31, 2019

Reporting Summary

Nature Research wishes to improve the reproducibility of the work that we publish. This form provides structure for consistency and transparency in reporting. For further information on Nature Research policies, see Authors & Referees and the Editorial Policy Checklist.

Statistics

For all statistical analyses, confirm that the following items are present in the figure legend, table legend, main text, or Methods section.

n/a	Confirmed	
☐	☒	The exact sample size (*n*) for each experimental group/condition, given as a discrete number and unit of measurement
☐	☒	A statement on whether measurements were taken from distinct samples or whether the same sample was measured repeatedly
☐	☒	The statistical test(s) used AND whether they are one- or two-sided<br>*Only common tests should be described solely by name; describe more complex techniques in the Methods section.*
☐	☒	A description of all covariates tested
☐	☒	A description of any assumptions or corrections, such as tests of normality and adjustment for multiple comparisons
☐	☒	A full description of the statistical parameters including central tendency (e.g. means) or other basic estimates (e.g. regression coefficient) AND variation (e.g. standard deviation) or associated estimates of uncertainty (e.g. confidence intervals)
☐	☒	For null hypothesis testing, the test statistic (e.g. *F*, *t*, *r*) with confidence intervals, effect sizes, degrees of freedom and *P* value noted<br>*Give P values as exact values whenever suitable.*
☒	☐	For Bayesian analysis, information on the choice of priors and Markov chain Monte Carlo settings
☒	☐	For hierarchical and complex designs, identification of the appropriate level for tests and full reporting of outcomes
☒	☐	Estimates of effect sizes (e.g. Cohen's *d*, Pearson's *r*), indicating how they were calculated

Our web collection on statistics for biologists contains articles on many of the points above.

Software and code

Policy information about availability of computer code

Data collection	*MiSeq Control Software V2.6.2<br>*MiSeq RTA V.1.18.54
Data analysis	*R v3.4.4<br>*Dropseq tools v1.13<br>*MIGEC 1.2.9<br>*MiXCR 2.1.12<br>*Agilent Genomic Workbench software version 7.0.4.0<br>*CIMPL (Common Insertion site Mapping PLatform)<br>*TAPDANCE (Transposon Annotation Poisson Distribution Association Network Connectivity Environment)<br>*RMA<br>*limma<br>*Trimmomatic v0.36<br>*Flash v.1.2.11<br>*BBMap<br>*survival v2.41-3<br>*custom script for generation of clonality network plots

For manuscripts utilizing custom algorithms or software that are central to the research but not yet described in published literature, software must be made available to editors/reviewers. We strongly encourage code deposition in a community repository (e.g. GitHub). See the Nature Research guidelines for submitting code & software for further information.

Data

Policy information about availability of data

All manuscripts must include a data availability statement. This statement should provide the following information, where applicable:

- Accession codes, unique identifiers, or web links for publicly available datasets
- A list of figures that have associated raw data
- A description of any restrictions on data availability

Sequence data have been deposited at EBI European Nucleotide Archive under accession number PRJEB31032.

Field-specific reporting

Please select the one below that is the best fit for your research. If you are not sure, read the appropriate sections before making your selection.

☒ Life sciences ☐ Behavioural & social sciences ☐ Ecological, evolutionary & environmental sciences

For a reference copy of the document with all sections, see nature.com/documents/nr-reporting-summary-flat.pdf

Life sciences study design

All studies must disclose on these points even when the disclosure is negative.

Sample size	No sample size calculations were performed. Sample sizes were determined based on previous experiments.
Data exclusions	No data were excluded.
Replication	For CRISPR/Cas-based validation of candidate genes, biological replicates (Phip-sgRNA: n=7; Rfx7-sgRNA: n=5, Trp53-sgRNA: n=11, Trp53-shRNA: n=4, NT-sgRNA: n=9) were used. Cell culture based competition assays were performed in duplicates. qPCRs were performed in triplicates.
Randomization	Mice were allocated in cohorts based on genotype. No randomization was performed.
Blinding	Investigators performing necropsies and histopathological analyses were blinded to the genotype.

Reporting for specific materials, systems and methods

We require information from authors about some types of materials, experimental systems and methods used in many studies. Here, indicate whether each material, system or method listed is relevant to your study. If you are not sure if a list item applies to your research, read the appropriate section before selecting a response.

Materials & experimental systems

n/a	Involved in the study	
☐	☒	Antibodies
☐	☒	Eukaryotic cell lines
☒	☐	Palaeontology
☐	☒	Animals and other organisms
☒	☐	Human research participants
☒	☐	Clinical data

Methods

n/a	Involved in the study	
☒	☐	ChIP-seq
☐	☒	Flow cytometry
☒	☐	MRI-based neuroimaging

Antibodies

Antibodies used	*B220/CD45R (RA3-6B2; R&D Systems; 1:40 dilution)<br>*CD138 (281-2; BD Biosciences; 1:50 dilution)<br>*CD3 (A0452; DAKO; 1:100 dilution)<br>*myeloperoxidase (A0398; DAKO; 1:100 dilution)<br>*Ki-67 (RM-9106-S1; Thermo Fisher Scientific; 1:200)<br>*Bcl6 (sc-858; Santa Cruz Biotechnology; 1:50)<br>*Irf4 (sc-6059; Santa Cruz Biotechnology; 1:100<br>*Pten (M362729-2; Agilent; 1:150 dilution)
Validation	All antibodies were validated for specificity and sensitivity using positive and negative control samples.

Eukaryotic cell lines

Policy information about cell lines

Cell line source(s)	*HT: ATCC<br>*HEK293T: ATCC<br>*RIVA: DSMZ
Authentication	Cell lines were not authenticated.
Mycoplasma contamination	All cell lines were tested negatively for mycoplasma contamination.
Commonly misidentified lines<br>(See ICLAC register)	No commonly misidentified cell lines were used.

Animals and other organisms

Policy information about studies involving animals; ARRIVE guidelines recommended for reporting animal research

Laboratory animals	*ITP2-M: this study<br>*ITP-1C: this study<br>*Rosa26Cas9 (Gt(ROSA)26Sortm1(Cas9)Rad): this study<br>*Rosa26PB: Rad et al. Science 2010<br>*Blmm3: Luo et al. Nature Genetics 2000<br>*Eμ-myc: Adams et al. 1985<br><br>Mice were maintained on a mixed C57BL/6J x 129S1/SVImJ x FVB/NJ background.<br>Female as well as male mice were used. Mice were aged for tumor development.<br>Female C57BL/6J mice (~10 weeks) were used for transplantation experiments.<br>Eμ-Myc;Rosa26Cas9 E13.5 embryos were used to source fetal liver cells.
Wild animals	The study did not involve wild animals.
Field-collected samples	The study did not involve samples collected from the field.
Ethics oversight	All animal experiments were carried out in compliance with the requirements of the European guidelines for the care and use of laboratory animals and were approved by the local authorities.

Note that full information on the approval of the study protocol must also be provided in the manuscript.

Flow Cytometry

Plots

Confirm that:

☒ The axis labels state the marker and fluorochrome used (e.g. CD4-FITC).

☒ The axis scales are clearly visible. Include numbers along axes only for bottom left plot of group (a 'group' is an analysis of identical markers).

☒ All plots are contour plots with outliers or pseudocolor plots.

☒ A numerical value for number of cells or percentage (with statistics) is provided.

Methodology

Sample preparation	Cells (HSPCs, HT and RIVA cells) were washed 2x with PBS before FACS analysis. HT and RIVA cells were fixed with 2% PFA.<br>No antibody stainings were performed.
Instrument	*FACSCanto II flow cytometer (BD Biosciences)<br>*CytoFLEX S (Beckmann Coulter)<br>*FACSARIA III cell sorter (BD Biosciences)
Software	*FlowJo (Tree Star Inc.)<br>*Kaluza Software (Beckmann Coulter)
Cell population abundance	HSPCs: 20-30% of cells were GFP positive.<br>HT and RIVA cells: 20-40% of cells were BFP positive.

Gating strategy

FSC/SSC gating was performed to exclude cell debris.
Gating for DAPI negative cells was performed to discriminate viable cells from dead cells (DAPI positive).
HT and RIVA cells: Fraction of transduced cells was determined by gating for BFP positive cells.
HSPCs: Fraction of transduced cells was determined by gating for GFP positive cells.

☒ Tick this box to confirm that a figure exemplifying the gating strategy is provided in the Supplementary Information.
